# Robotically equipped 3D hybrid operating room in spine and trauma surgery – updated experience in 359 patients and 2080 screws

**DOI:** 10.1007/s11701-026-03898-1

**Published:** 2026-09-04

**Authors:** Dominik M. Haida, Peter Mohr, Thorsten Möhlig, Friedemann Meiswinkel, Mircea-Andrei Ungureanu, Thorsten Enk, Marc Hanschen, Stefan Huber-Wagner

**Affiliations:** 1 Technical University of Munich, School of Medicine and Health, TUM Klinikum, Rechts der Isar, Department of Trauma Surgery, Ismaninger Straße 22, 81675 Munich, Germany; 2 DIAK Klinikum Landkreis Schwäbisch Hall, Department of Trauma, Spine & Orthopaedic Surgery, Level-I Trauma Centre, Spine Centre (DWG), Diakoniestraße 10, 74523 Schwäbisch Hall, Germany; 3 DIAK Klinikum Landkreis Schwäbisch Hall, Radiation Protection, Diakoniestraße 10, 74523 Schwäbisch Hall, Germany; 4 DIAK Klinikum Landkreis Schwäbisch Hall, Department of Neurosurgery, Diakoniestraße 10, 74523 Schwäbisch Hall, Germany

**Keywords:** Robotics, Navigation, Robotic arm, Hybrid OR, Pelvis, Acetabulum, Mixed-Reality, Learning curve, Neurosurgery, Cone beam CT (CBCT)

## Abstract

Modern techniques, such as robotics and navigation, alongside cutting-edge imaging solutions like cone beam CT imaging, are playing an increasingly prominent role in spine and trauma surgery within so-called Hybrid ORs. This study aims to contribute to the ongoing scientific discussion surrounding accuracy rates and learning curves in the utilisation of a modern Hybrid OR. In a Level 1 trauma centre, a 3D Navigation-Hybrid OR was implemented, and a prospective data collection was launched (March 2022 - June 2025). Analysis was performed retrospectively. The Hybrid OR consists of a robotic cone beam CT “Loop-X”, a robotic arm “Cirq Arm System”, a navigation unit “Curve Navigation System”, a wall-mounted monitor “BUZZ” and Elements Viewer 3D mixed reality glasses for planning (Brainlab, Munich, Germany). Intervention areas included the entire skeletal system, encompassing the spine, pelvis (pelvic ring and acetabulum), and extremities, with various indications, primarily trauma (fractures) and oncology (tumours). The focus of this study is to assess accuracy rates and learning curves. In *n* = 359 patients, 2080 screws were inserted. 1881 (90.4%) spinal screws were inserted with an accuracy rate (GR-A & GR-B) of 98.5% (CI95%: 97.9% − 99.0%). A total of 199 (9.6%) non-spinal screws were inserted with an accuracy rate of 99.0% (CI95%: 97.6% − 99.9%). The overall accuracy rate of screw placement was therefore 98.5% (CI95%: 98.0% – 99.0%). Minimally invasive techniques were used in 243 (67.7%) cases and the robotic arm was utilised in 271 (75.5%) cases. Subgroup analyses revealed a significant learning curve (*p* < 0.001) and a significant reduction in operation times over time (*p* = 0.008). Wound infection occurred in 10 (2.8%) cases, and 17 (4.7%) patients died during hospitalisation. This study demonstrates that a 3D-Navigation Hybrid OR can be successfully integrated into the clinical routine of a level 1 trauma centre. It shows promising results for interventions on the entire skeletal system, with consistently high accuracy rates and low complication rates. Furthermore, it highlights a significant learning curve and a significant reduction in operation times over time.

## Introduction

The field of surgery is continually evolving, with innovations dramatically changing the daily work of surgeons. Over time, the aims of cutting-edge technologies have changed. Today, many surgical specialities focus on robotic surgery [1–5]. For patients, the potential benefits include improved outcomes, particularly through enhanced precision, which contribute to the safety of operations, as well as more options for minimally invasive procedures. These factors collectively can lead to potential improved patient outcomes [6–9].

However, these technologies also offer considerable advantages for surgeons and the entire Operating Room (OR) team. In trauma and spine surgery, intraoperative imaging with hazardous radiation is often unavoidable. Modern Hybrid Operating Room (Hybrid OR) setups, which combine robotics with image guidance (“navigation”) and modern imaging solutions such as Cone beam computed tomography (CBCT), offer practical solutions for reducing radiation exposure [10, 11]. Furthermore, compared with conventional techniques, robotically assistance has the potential to reduce physical strain and fatigue in surgeons [11–14]. This can enable surgeons to maintain a consistently high level of performance throughout the entire operation, potentially creating a win-win situation for both surgeons and patients. Nevertheless, several important factors must be considered. An example is the increasing economisation of healthcare systems and the high patient volumes, which make operation times and learning curves key areas requiring investigation.

This retrospective study *aims* to present and discuss experiences with a modern 3D-Hybrid-Navigation OR in a level-1-trauma centre. The overall focus of this study is to evaluate the experience with the use of a modern Hybrid OR at the entire skeletal system, with particularly analysing accuracy rates and learning curves. The authors consider it of the utmost importance to contribute to the ongoing scientific discussion by focusing on achievable accuracy rates and analysing learning curves in the application of a modern Hybrid OR.

## Methods and materials

The authors emphasise that an article based on the findings with the initial 210 patients was published in September 2024 [11]. The present manuscript extends this earlier publication by including a total of 359 cases. The analyses were conducted analogously to those in the previous manuscript to reassess changes and previous findings. The number of operating surgeons was expanded and new intervention areas were explored. In addition, a learning-curve analysis was feasible and has been included. For certain aspects of the methods, such as the workflow, we refer to this previous publication [11].

With the implementation of the 3D-Navigation Hybrid-OR at the author’s hospital, a prospective data collection was launched. Patients who underwent surgery from the implementation of the 3D-Navigation Hybrid-OR in March 2022 through June 2025 were included consecutively and prospectively. The data was analysed retrospectively. Inclusion in this study required a postoperative computed tomography (CT) scan. Between March 2022 and September 2024, the cases were operated by three consultants. From September 2024 onwards, up to the time of the analysis, the number of operating consultants was increased to seven. The variety of indications included mainly trauma (fracture), oncological diseases, spondylodiscitis, degenerative diseases and rheumatic diseases. Intervention areas were the spine, pelvis (acetabulum and pelvic ring) and extremities. Specific technical descriptions for different intervention sites were published along with a series of videos [15–18].

The 3D-Navigation Hybrid-OR consist of the following parts: robotic 3D cone beam CT “Loop-X”, navigation unit “Curve Navigation System”, robotic arm “Cirq Arm System”, wall-mounted monitor “BUZZ” and Elements Viewer 3D mixed reality glasses for planning (Brainlab, Munich, Germany).

The authors’ institute is a hospital certified as a Level I trauma centre and a geriatric trauma centre by the German Trauma Society (DGU).

Ethical approval was obtained from the Landesärztekammer Baden-Württemberg (Stuttgart, Germany), with reference numbers F-2024-037 and F-2024-037_1. Registration of this study was conducted retrospectively in the German Clinical Trials Register on 9 July 2024, with the identification number DRKS00034551.

### Setting

In comparison to our analysis of the first 210 patients, several team-related aspects changed. While an employee from the manufacturer is still present at every operation, the number of main surgeons operating independently in the Hybrid OR has increased from three to seven consultants. All have years of experience in conventional and hand navigation techniques for the respective intervention areas. The transition to independent surgery was achieved through theoretical training and continuous assistance during operations in the hybrid operating theatre. To maintain competence in the use of robotics and navigation with its hardware and software, we regularly hold refresher and revision courses for the entire OR team.

The instruments used for the operations at the spine were mainly Solera (Medtronic, Meerbusch, Germany) and Symphony (DePuy Synthes, Umkirch, Germany). For operations performed at the pelvis and extremities, osteosynthesis material from Königsee Implantate (Allendorf, Germany), DePuy Synthes (Umkirch, Germany), and KLS Martin (Tuttlingen, Germany) were utilised.

Patients which arrived in the hospital in the trauma room, were categorised as trauma room patients. The Injury Severity Score (ISS) of these patients was collected from trauma room protocols and the Trauma Registry documentation for the Trauma registry (TR-DGU) of the German Trauma Society (DGU).

### Data acquisition and collection

Descriptive and demographic data were collected with the assistance of accounting management from various sources, including clinical documentation, letters of release, trauma room protocols, surgical reports, and operation documentation [11]. For grading of the screw positions, preoperative planning and postoperative CT scans were used.

### Radiation data

The basic methodology is analogous to that of the previous publication; for technical details regarding the CBCT, we refer readers to that publication [11]. CBCT 3D scan data was collected from the picture archiving and communication system (PACS) including a radiation dose structured report (RDSR) in digital Imaging and Communications (DICOM) format [11]. The unit of the dose information is cGy cm^2^ (dose area product “DAP”). Radiation data were available for 353 of the 359 patients, with one additional failed data transfer (due to an unknown reason) compared to the first analysis, resulting in a total of six. These patients were excluded from the radiation data analysis, as in the previous manuscript. Since the last analysis, regular software updates have been carried out with new (primarily higher-dose) protocols now available. The focus of this analysis is on 3D CBCT data, as the authors consider this to be the primary point of interest. For this reason, only 3D scan CBCT data were collected and analysed from patient 211 onwards, as it presents the main source of radiation, as analysed in the past [11].

### Data analysis

#### General

For this manuscript, data from the initial publication and the newly acquired data were reanalysed together, forming a new study population.

In this manuscript, the term “average” refers to the arithmetic mean.

Statistical analyses were performed using SPSS Statistics software version 31.0 (IBM Corp., Armonk, N.Y., USA).

### Grading of screw accuracy

Screws inserted at the spine (C1-S1), so-called “spinal screws”, were graded according to the Gertzbein and Robbins classification (GR) for pedicle screws [11, 19, 20]. The grading was carried out independently by the two researchers, SHW and DMH.

- **GR-A**: no breach of the pedicular cortex, complete intrapedicular screw
- **GR-B**: < 2 mm breach
- **GR-C**: 2–4 mm breach
- **GR-D**: < 6 mm breach
- **GR-E**: > 6 mm breach [11, 19, 20].

For some interventions, larger screws diameters than pedicular diameters were required for biomechanical stability. In these cases, screws were graded GR-A when the screws penetration of the pedicle was centred in its exact geometry [11].

By definition, GR-A and GR-B positions are considered as an accurate/optimal screw placement, whereas GR-C, -D, -E screws are regarded as an inaccurate/suboptimal screw placement [11, 19, 20].

For screws inserted not at the spine but at other areas (pelvis and extremities), so-called “non-spinal screws”, there is no existing classification for assessment of the screw accuracy. For this reason, the grading of the screws was carried out by the comparison of the preoperative screw planning with the postoperative CT scan, in which the postoperative screw position was assessed. When the screw position was in accordance with the preoperative screw planning (corresponding screws trajectory) and without an intentional cortical breach, the screw was considered as “optimally placed” (comparable to GR-A or GR-B). If the screw position was not in accordance with the planning, the screw was considered as “suboptimally placed” (comparable to GR-C, GR-D and GR-E).

The reviewers were not blinded to the patients and their characteristics during screw assessment. In case of disagreements, a consensus-based assessment was performed. Since 2023 postoperative CT images have been acquired using the high-resolution spectral 128-slice CT scanner SOMATOM X.ceed (Siemens Healthineers, Erlangen). Interrater reliability between the two reviewers SHW and DMH was assessed across the entire cohort of 359 patients using unweighted Cohen`s Kappa with the help of cross tabulation. Interrater reliability was interpreted according to Landis and Koch [21].

### Operation time analysis

Operation time was defined as the time from incision to suture (incision-to-suture time). In the following, incision-to-suture time is referred to as operative or operation time. The average operative time was calculated for different subgroups for better comparability. In Addition to the analysis of 210 patients, the operative time for spinal interventions was described to enhance comparability with studies that only included spinal screw placement.

To examine potential changes in operative times, an explorative subgroup analysis of all dorsal stabilisations (*n* = 188) was conducted. Given the right-skewed distribution observed in the descriptive analysis, the Mann-Whitney-U-Test for independent samples was considered more appropriate than the t-test. The subgroup was divided into two equal groups, each with *n* = 94 patients. Following this, a Mann-Whitney-U-Test was then performed using the statistics software. The null hypothesis (*H*_*0*_) stated that there were no differences in operative times between the two groups, while the alternative hypothesis (*H*_*1*_*)* stated that there were differences in the operative times between the groups. The significance level was set at α = 0.05.

### Subgroup learning curve analysis - operative time

In the initial analysis of 210 patients, a learning curve was not visible for all interventions due to the variety of interventions performed. Therefore, for the analysis of 359 patients in this manuscript, the authors focused on an explorative subgroup analysis with comparable types of interventions. The decision was made to perform a subgroup analysis for all four-segment dorsal stabilisations without laminectomies and decompressions performed. The reason, therefore, was that four-segment dorsal stabilisations were the most commonly performed procedure in our dataset, with 87 patients operated. All patients in the subgroup were assigned a number, ranging from 1 to 87, based on their order of operation in the Hybrid OR. A scatter plot of all 87 interventions with a logarithmic trendline was inserted, which showed a learning curve at a first glance. The natural logarithmic trendline was selected based on the assumption that learning of the operations occurred rapidly in the initial operations, followed by a plateau at a certain point.

Accuracy rates and 95% confidence intervals of the subgroups were calculated separately. Further investigation included, with the help of R^2^ and the natural logarithmic trendline of the scatter plot for all 87 patients ($\:y\:=\:-\mathrm{18,67}ln\left(x\right)\:+\:\mathrm{235,36}$), the calculation of a breaking point. Prior to the analysis, the authors defined the breaking point as the operation number in the subgroup at which the reduction of operative time compared to the previous procedure fell below one minute. For the early cases up to the breaking point (1–19) and for the entire subgroup (1–87), a natural logarithmic regression was performed to examine the decrease in operative time across successive procedures. For the regression, the consecutive case numbers were first transformed using the natural logarithm (ln). Following this, a standard linear regression was performed on the transformed case numbers (ln of the case numbers) as the independent variable and operative time as the dependent variable. This approach is mathematically equivalent to a natural logarithmic regression, which is not directly available in the used statistical software. The null hypothesis (*H*_*0*_) stated that there was no decrease in operative time over time (no learning curve), while the alternative hypothesis *(H*_*1*_) stated that there was a decrease in operative time (learning curve) over time. The significance level was set at *α* = 0.05. The accuracy rates of the subgroups were calculated analogues to the calculations presented in Table 9 under “overall accuracy”.

## Results

### Descriptive and demographic data

**Table 1:** Descriptive and demographic data

Variable	Value
Number of patients	359
Gender: Number of Male : Female (%)	198 : 161 (55 : 45)
Average age at time of intervention in years (Standard Deviation (± SD))	68.2 (± 16.4)
Number of infections (%)	10 (2.8%)
Number of in-hospital mortality (%)	17 (4.7%)

*BMI: body mass index*

### Trauma room data

**Table 2:** Trauma room data

Variable	Value
Number of trauma room patients (%)	47 (13.1%)
Number of trauma room patients with an ISS ≥ 16 (%)	33 (9.2%)
Average ISS of trauma room patients (± SD)	21.9 (15.8)

*ISS: injury severity score*

### Patient characteristics and Trauma room data

A total of *n* = 359 patients received treatment in the 3D-Navigation-Hybrid-OR. The cohort includes 198 (55%) male patients and 161 (45%) female patients. The average age at the time of intervention was 68.2 (± 16.4) years. Postoperatively, 10 (2.8%) infections occurred according to the classification for surgical site infections from the Centers for Disease Control and Prevention (CDC) [22]. The number of deaths during hospitalisation was 17, resulting in an in-hospital mortality rate of 4.7%. Out of 359 patients, 47 were trauma room patients, of whom 33 (9.2%) had an ISS ≥ 16. The average ISS of all trauma room patients was 21.9 with a standard deviation of 15.8 (Tables 1 and 2).

### Diagnoses/Indications

**Table 3:** Analysis of Diagnoses and indications with percentage share

Diagnoses / Indications (partially overlapping)	Number of patients (%)
Fracture	311 (86.6%)
Osteoporosis	115 (32.0%)
Tumour/Oncology	72 (20.1%)
Deformity	24 (6.7%)
Degenerative diseases	18 (5.0%)
Spondylodiscitis	14 (3.9%)
Morbus Bechterew	6 (1.7%)
Rheumatic diseases	2 (0.6%)
Preoperative dural leakage	2 (0.6%)

### Intervention areas

**Table 4:** Analysis of all intervention areas with percentage share

Intervention areas (partially overlapping)	Number of patients (%)
Cervical spine	54 (15.0%)
Thoracic spine	170 (47.4%)
Lumbar Spine	141 (39.3%)
Pelvic ring	81 (22.6%)
Extremities	23 (6.4%)
Acetabulum	17 (4.7%)
Maxillofacial	2 (0.6%)

### Operation indications and areas of intervention

The three main diagnoses/indications (partially overlapping) were fractures in 311 (86.6%) cases, osteoporosis in 115 (32.0%) cases and oncology (tumour) in 72 (20.1%) cases (Table 3). The 359 interventions were performed, partially overlapping, throughout the entire musculoskeletal system. These included interventions involving the cervical spine in 54 (15.0%) cases, the thoracic spine in 170 (47.4%), the lumbar spine in 141 (39.3%), the pelvic ring in 81 (22.6%), the acetabulum in 23 (6.4%), the extremities in 17 (4.7%) and the maxillofacial region in 2 (0.6%) (Table 4).

### Key surgical data

**Table 5:** Key surgical data with sum and percentage share.

Operative factors	Number of patients (%)
Robotic arm in use	271 (75.5%)
MIS	243 (67.7%)
Cement augmentation	187 (52.1%)
Microsurgical technique	87 (24.2%)
Laminectomies/decompressions	66 (18.4%)
Kyphoplasty	19 (5.3%)
TLIF	14 (3.9%)
Thermal ablation	9 (2.5%)
Preoperative embolization	10 (2.8%)

*MIS: minimally invasive surgery. TLIF: transforaminal lumbar interbody fusion*

### Intraoperative data

In 271 (75.5%) cases, the robotic arm was used. Minimally invasive surgery techniques (MIS) were used in 243 (67.7%) cases. Cement augmentation of the screws was required in 187 (52.1%) cases, and a microsurgical technique (using magnifying glasses, microscope or an exoscope) was applied in 87 (24.2%) cases. In the case of 66 (18.4%) patients, a decompression of the spinal cord was necessary (Table 5).

### Operative time analysis

The average incision-to-suture time for all interventions was 201 (± 115) minutes with a median incision-to-suture time of 175 min. For all operations where no laminectomies and decompressions were performed, the incision-to-suture time was shorter, with 169 (± 75) minutes, with a median of 158 min (Table 6).

Subgroup analysis of a learning curve for four-segments dorsal stabilisations without laminectomies and decompressions: Logarithmic regression.

**Table 6:** Analysis of the operation times

Duration of operations	Time in minutes (± SD)
Average for all interventions	201 (± 115) Range (minimum (min.)/maximum(max.)): 35–810
Median for all interventions	175
Average for interventions without laminectomies and decompressions	169 (± 75) Range (min./max.): 35–456
Median for interventions without laminectomies and decompressions	158
Average for interventions with laminectomies and decompressions	346 (± 146) Range (min./max.): 133–810
Median for interventions with laminectomies and decompression	299
Average for spinal interventions	229 (± 116)
Median for spinal interventions	204

**Fig. 1:**
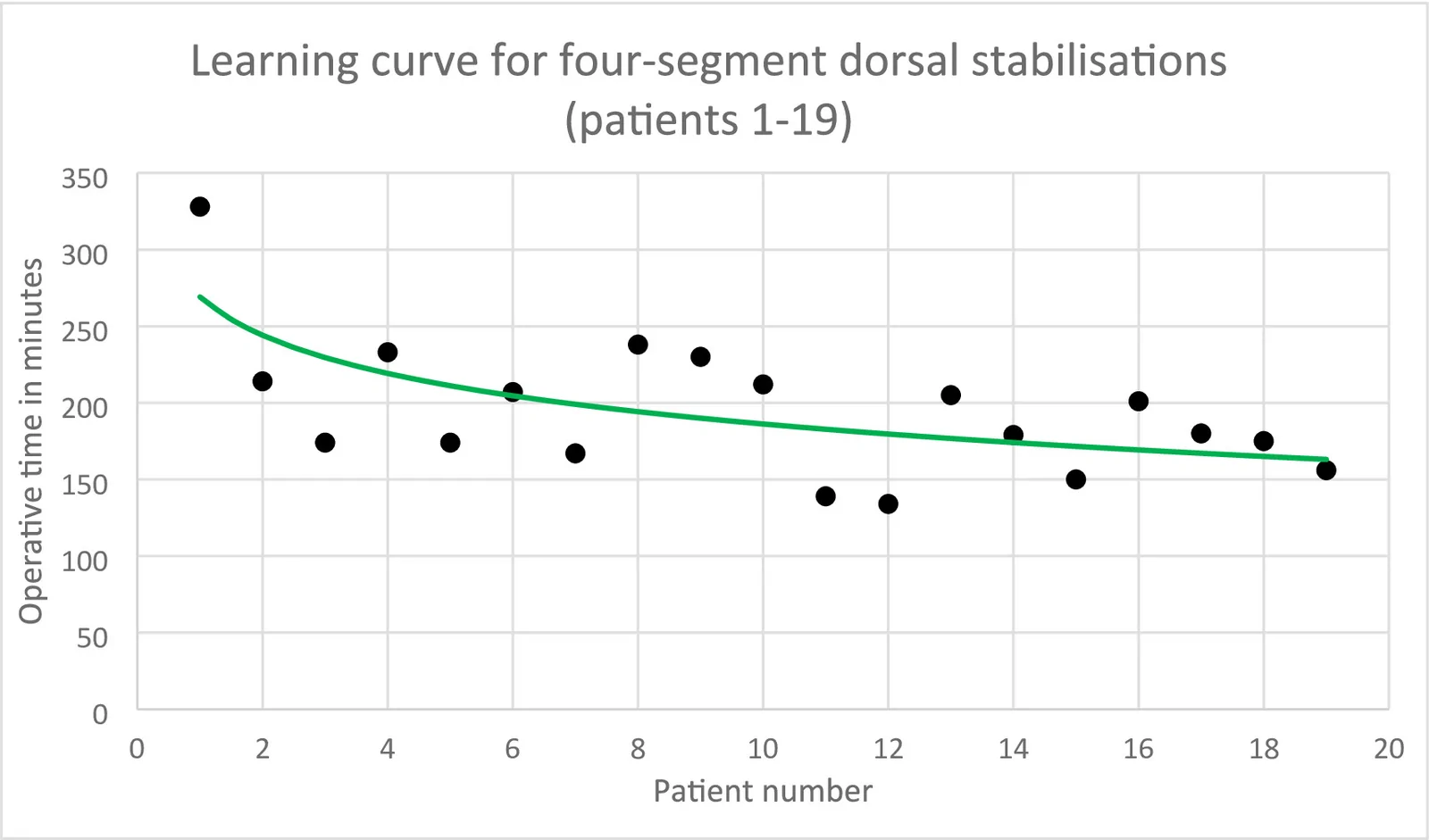
Scatter plot for four-segment dorsal stabilisations until the breaking point with a logarithmic trendline (green line)

**Fig. 2:**
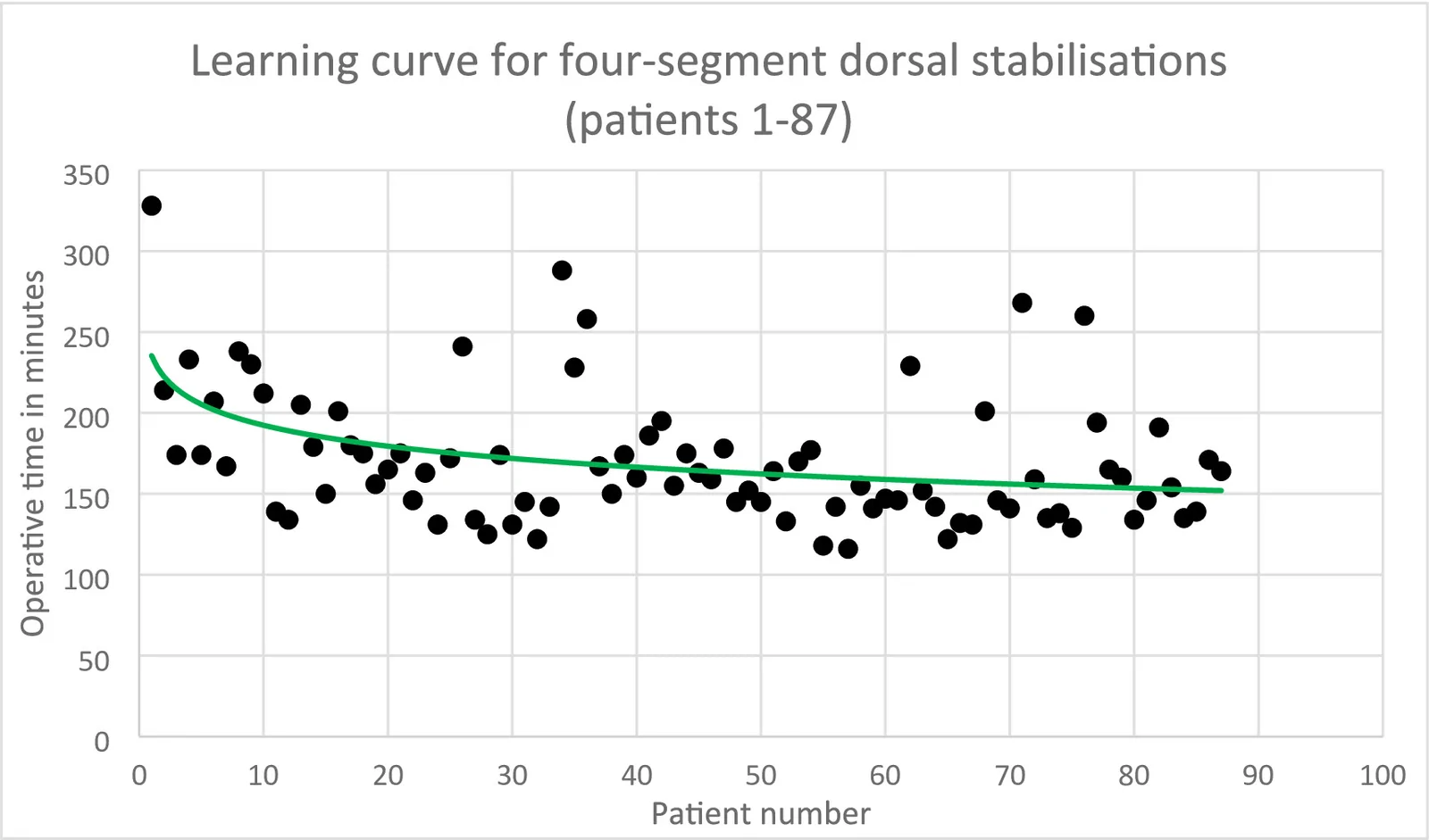
Scatter plot for all four-segment dorsal stabilisations with a logarithmic trendline (green line)

The breaking point was identified at around case 19, where the reduction of operative time per case fell below one minute. Accuracy rates of the subgroups were 98.6% and 98.4%. The average operative time was 195 (± 45) minutes for the first 19 patients and 170 (± 41) minutes for all 87 patients in the subgroup analysis. The median operative time was 180 min for the breaking point group, which was 20 min higher than for the entire subgroup. The statistical analysis of the breaking point group revealed a significant change in the operative time, with a *p*-value of 0.003 and an adjusted R^2^ value of 0.388. The analysis of the entire subgroup revealed a highly significant change in operative time, with a *p*-value of < 0.001 and an adjusted R² of 0.170 (Table 7; Figs. 1 and 2). With the significance level set at *α* = 0.05, the null hypothesis, which stated that there is no decrease in the operative time over time, was rejected for both groups. The alternative hypothesis (H_1_), which stated a significant decrease in the operative time over time, was supported by the results of the logarithmic regression for both groups.

**Table 7:** Subgroup analysis of a learning curve for all four-segments dorsal stabilisations with *n* = 87 patients using logarithmic regression. The table shows a comparison of patients 1–19, treated before the learning curve breaking point with patients of the entire subgroup (patients 1–87) B: change in operative time per logarithmic increase in patient number

	Data for patients 1–19 (Patients until breaking point)	Data for patients 1–87 (Entire subgroup)
Number of patients	19	87
Screws inserted	143	670
Accuracy rate (CI95%)	98.6% (96.7%-99.0%)	98.4% (97.4%-99.3%)
Average operative time in minutes (± SD) (Range: min.-max.)	195 (± 45) (134–328)	170 (± 41) (116–328)
Median operative time in minutes	180	160
IQR (Q3-Q1) in minutes	47	28
**Statistical analysis for operative time**		
Adjusted R^2^	0.388	0.170
“B” = change of Incision-to-suture time in minutes	–36	–19
t-Value for change in operative time	–3.523	–4.321
*p*-Value for change in operative time	0.003	< 0.001

Subgroup analysis of operative times for all dorsal stabilisations: Mann-Whitney-U-Test.

Subgroup analysis was performed for all dorsal stabilisations with *n* = 188 patients (Table 8). The Mann-Whitney U-Test revealed a statistically significant difference in the incision-to-suture times between both groups (U = 3424; Z = 2.548; *p-value* = 0.008). Since the *p*-value was below the set significant level of *α* = 0.05, the null hypothesis (*H*_*0*_) was rejected, and the alternative hypothesis (*H*_*1*_) was supported by the results of the analysis.

**Table 8:** Subgroup analysis of all dorsal stabilisations with *n* = 188 patients using the Mann-Whitney-U-Test.

Group	Average operative time in minutes (± SD)	Median operative time in minutes (IQR)	Test statistic “U”	Standardised test statistic “Z”	*p*-value
Case 1–94 (*n* = 94 patients)	190 (± 66)	174 (88)	3424	2.548	0.008
Case 95–188 (*n* = 94 patients)	170 (± 60)	160 (90)			

*IQR: Interquartile Range*

### Radiation

**Table 9:** Radiation given in dose area products (DAPs) at Loop-X 3D scans for spinal and non-spinal patients. DAPs are given in units of cGy cm^2^

Radiation at Loop-X 3D scans for 254 spinal patients	Number (± SD) / DAP (± SD)
Number of 3D-scans	2.20 (± 0.61)
Dose area product per 3D scan	1194 (± 825)
Dose area product of all 3D scans	2631 (± 2205)
**Radiation at Loop-X 3D scans for 99 non-spinal patients**	
Number of 3D-scans	1.84 (± 0.62)
Dose area product per 3D scan	1262 (± 809)
Dose area product of all 3D scans	2321 (± 1759)

Radiation data were available for 353 of the 359 patients, with one additional failed data transfer (due to an unknown reason) compared to the first analysis, resulting in a total of six. These patients were excluded from the radiation data analysis, as in the previous manuscript. The average number of 3D scans was 2.20 (± 0.61) for spinal interventions and 1.84 (± 0.62) for non-spinal interventions. The average dose area product of all 3D scans was 2631 (± 2205) cGy cm² for spinal interventions, which was higher than that of non-spinal interventions, with an average dose area product of 2321 (± 1759) cGy cm². (Table 9).

### Hospitalisation data

**Table 10:** Details about the hospital length of stay and ICU stays

Hospital length of stay & ICU stays	Time in days (± SD) / Number of patients (%)
Average hospital length of stay for all interventions	16.8 (± 11.0)
Average hospital length of stay for interventions without laminectomies and decompressions	15.4 (± 9.0)
Average hospital length of stay for interventions with laminectomies and decompression	23.1 (± 15.6)
Total number of patients with an ICU stay	121 (33.7%)
Average length of ICU stays for all interventions	1.3 (± 4.0)
Number of patients with an ICU stay for interventions without laminectomies and decompressions	88 (24.5%)
Average length of ICU stays for interventions without laminectomies and decompressions	1.2 (± 4.3)
Number of patients with an ICU stay for interventions with laminectomies and decompressions	34 (9.5%)
Average length of ICU stays for interventions with laminectomies and decompression	1.5 (3.2)

The Average hospital length of stay for all 359 patients included in this study (all interventions) was 16.8 (± 11.0) days. For interventions without laminectomies and decompressions, the average hospital length of stay was 15.4 (± 9.0) days, which was more than seven days shorter than for patients who underwent interventions with laminectomies and decompressions, at 23.1 (± 15.6) days. In total, 121 (33.7%) had postoperative ICU stays (Table 10).

### Accuracy

**Table 11:** Analysis of the accuracy for spinal and non-spinal interventions

Variable	Number (%) / Number (± SD)
Total number of screws inserted	2080
Spinal screws inserted	1881 (90.4%)
Non-spinal screws inserted	199 (9.6%)
Average number of inserted screws per patient	5.8 (± 3.5)
Average bridged segments (spine)	3.9 (± 1.5)
Revisions	0 (0%)
Grading of spinalscrews according to the Gertzbein and Robbins (GR) classification [11, 19, 20]	
GR-A	1604 (85.2%)
GR-B	248 (13.2%)
GR-C	21 (1.1%)
GR-D	6 (0.3%)
GR-E	2 (0.1%)
GR-C, GR-D, GR-E with neurological symptoms	0 (0%)
Suboptimally placed	29 (1.5%)
Accuracy spinal screw insertion:	98.5% CI95%: 97.9% − 99.0%
Grading ofnon-spinal screws	
Optimally placed	197 (99.0%)
Suboptimally placed	2 (1.0%)
Accuracy of non-spinal screw insertion:	99.0% CI95%: 97.6% − 99.9%
Overall malposition rate	
Overall malposition rate:	1.5% CI95%: 1.0% − 2.0%
Overall accuracy	
Overall accuracy:	98.5% CI95%: 98.0% – 99.0%

- *a) Accuracy of spinal screw placement* With 1881 (90.4%) spinal screws inserted and 5.8 (± 3.5) segments bridged on average, the accuracy for spinal screw placement was 98.5% (CI95%: 97.9% − 99.0%). However, 29 screws were suboptimal placed (GR-C: 21; GR-D: 6; GR-E: 2).
- *b) Accuracy of non-spinal screw placement* At the pelvis and extremities, 199 non-spinal screws were inserted, with 197 (99.0%; CI95%: 97.6–99.0%) screws optimally placed and 2 (1.0%) screws suboptimally placed.
- *c) Overall accuracy* Spinal Screw placement and non-spinal screw placement, with an average of 5.8 (± 3.5) screws inserted, result in an overall accuracy rate of 98.5% (CI95%: 98.0% – 99.0%). The revision was, with no revisions necessary, at 0% (Table 11).
- *d) Interrater reliability* The observed agreement rate between both screw graders was 87.4% in overall screw assessment. Cohen`s Kappa for agreement in screw assessment was calculated with 0.718 (CI95%: 0.687–0.749). According to Landis and Koch interpretation of Cohen`s Kappa, the agreement can be considered substantial.

## Discussion

As far as the authors are aware, the present study reports one of the largest cohorts to date, which underwent navigated and robotically assisted surgery in a 3D-Navigation Hybrid OR at the spine, pelvis, and extremities. A total of 2080 screws with an overall accuracy rate of 98.5% were inserted into 359 patients with a revision rate of 0%.

### Learning curve and operative time development

Compared with the previous data from 210 patients, the difference in operation times is minimal [11]. The comparison between groups shows that the differences in average operative times are within five minutes for all interventions, including those without laminectomies and decompressions performed. Given the variety of intervention types and the given standard deviation, this is entirely comprehensible for the authors. In contrast, the average operative time for interventions involving laminectomies and decompressions increased by 56 min. When interpreting this finding, it must be considered that laminectomies and decompressions can be very individual and complex, particularly in bleeding patients (severely injured patients or oncological patients). This is illustrated by the increase in the maximum duration of a single operation, from 557 to 810 min [11]. With these aspects in mind, the authors do not consider this change to be meaningful. The comparison of our spinal operative time with that reported by Pojskic et al. [23] for a comparable setup shows a similar averaged duration (when rounded to full minutes), but a higher standard deviation (+ 10 min) for our study.This is noteworthy, considering that interventions across the entire spine, the global spine, including cervical procedures, whereas Pojskic et al. focused exclusively on the thoracolumbar spine. The comparable times may be due to differences in imaging solutions, with an intraoperative CT (iCT) requiring more time within the workflow than a modern robotic cone beam CT. Comparisons with studies using other setups also show average operative times of comparable lengths, or differences that can be attributed to the variety of intervention sites or a strong focus on only a few intervention types [24–26].

A reduction in operation times over time was visible in our explorative subgroup analysis of 87 patients who underwent four-segment dorsal stabilisation without laminectomies or decompressions. The statistical analysis of patients up to the point at which the reduction in operative time per additional case operated fell below one minute (breaking point) confirms the presence of a significant learning curve (*p*-value = 0.003) in the early cases, characterised by larger time reductions. This is reflected in the coefficient “B”, which indicates a decrease in operative time of approximately 36 min per natural logarithmic increase in case number for the breaking point group (patients 1–19). The statistical analysis of the entire subgroup reveals a highly significant learning curve (*p*-value < 0.001), although with a lower adjusted R² of 0.170 compared with an R² of 0.388 for the initial 19 patients. This supports the suitability of a natural logarithmic learning curve, with the coefficient “*B*” indicating an average reduction of around 19 min per natural logarithmic increase in case number for the entire subgroup, suggesting that a plateau phase is reached in later cases.

The analysis of our cases is consistent with findings from other studies, which also reported a learning curve in the early phase. One study identified a breaking point close to ours [27–30]. Nevertheless, there are reports of longer learning curves with an extended initial learning phase described in a recently published meta-analysis reporting on learning curves regarding operative time and screw accuracy in robotically assisted pedicle screw placement [31]. Compared with our findings, the faster learning curve could be due to several factors including educative training prior the implementation and previous navigation experience Furthermore, methodological aspects of learning curve analyses should also be taken in consideration for the varying results. One example for this, is the statistical approach, as studies use different models to analyse learning curves [27, 32–35]. Methodological differences in statistical analyses therefore be may one of the key factors contributing to the reported variations.

The findings of the learning curve analysis align with the explorative subgroup analysis of all dorsal stabilisations in this study. To examine a change in operative times over time for all dorsal stabilisations, a methodologically different analysis with a Mann-Whitney-U-Test was performed. Therefore, 188 patients were split into two groups of 94 patients each, in the order they were operated and compared with the help of a Mann-Whitney-U-Test. This analysis revealed a significant difference (*p*-value = 0.008) in operative times between the two groups, with the second group (operated later) having significantly shorter operative times. This result suggests that learning has occurred at some point or over time, without specifying when it took place underlining the above demonstrated results.

However, the learning curve observed in our study may be confounded by the fact that experience had already been gained prior to the implementation of the Hybrid OR. It is also important to consider that operations performed between the cases included in the subgroup will presumably have reinforced the effect of the early learning curve, for the subgroup analysis may have reinforced the early learning effect, meaning that the true learning curve could in fact be flatter.

### Radiation

The discussion of radiation data remains complicated due to the use of different units and certain methodological factors. We begin by comparing the different results from our previous publication [11]. Overall, the average number of 3D scans increased minimally from 2.12 to 2.20 for spinal interventions and from 1.81 to 1.84 for non-spinal interventions. What has to be mentioned is the increase in the average DAP of all 3D scans at 359 patients compared to the values at 210 patients [11]. For spinal interventions, the increase was + 477 cGy cm^2^ per intervention (+ 178 cGy cm^2^ per 3D scan), and for non-spinal interventions, + 325 cGy cm^2^ (+ 161 cGy cm^2^ per 3D scan). The reasons for this will be multifaceted with the origin in patient characteristics, software updates with higher dose protocols, and due to procedures of different complexities [36, 37]. Different methodologies in reporting the radiation data and different units, make it complex to compare data from different studies. However, compared with the little available literature reporting with a comparable methodology, our findings are near or lower to the DAP values reported in spinal surgery with other cone beam CTs and fluoroscopy [11, 38, 39]. For the authors, it is important to reemphasise at this point, that the OR team leaves the room for 3D scans and therefore is not affected from the 3D scan radiation, which is by far the biggest source of radiation during operations in the Hybrid OR [11]. Due to the dependence on the exposed body part along with uncertainty and frequent changes of conversion factors, a calculation of effective doses is not included in this study [11, 40–42].

### Accuracy rate and accuracy learning curve

Latest evidence suggests that robotically assisted screw placement can be significantly superior to fluoroscopic screw placement in terms of accuracy when placing pedicle screws at the spine [9, 43–45]. These findings align with existing data on ultra-high accuracy rates achieved with the contribution of modern robotic systems [11, 26, 44, 46, 47]. The systematic review and meta-analysis by MacLean et al. [48] provide an overview of the accuracy rates achieved with the help of robotics, resulting in 25,054 screws inserted in 4670 patients. Reported accuracy rates for the respective robotic systems range from 94.2% to 98.2%. The accuracy rate of 94.2% originates from the first report by Pojskic et al. [41] and was the only study included in the meta-analysis for the specific robotic arm system used in this study [48, 49]. Both of our accuracy rates, at 210 and now at 359 patients, are well within the range of the reported 95% confidence intervals of the described robotic systems in the McLean et al. meta-analysis [48]. This reinforces the capabilities of the system that was used in this study. Since our last publication on 210 patients, only one new study was published regarding the accuracy rate of screw placement with a similar setup [23]. This publication is an update by Pojskic et al. [23] on their experience and developments with the system. The accuracy rate reported there is 93.9% (GR-A & GR-B) with 688 pedicle screws inserted at the thoracolumbar spine [23]. In comparison to their initial experience with an accuracy rate of 94.2% (GR-A & GR-B) and 70 screws inserted at the thoracolumbar spine, which we discussed in our prior publication, there was no improvement of the accuracy rate [49]. Compared to the accuracy rate of 98.5% achieved in this study, a difference of approximately four per cent demonstrates a substantial difference. This is particularly noteworthy, considering that in our study, more screws were placed in different anatomical regions using a similar workflow [11]. There may be various reasons for this. Technical differences between intraoperative CTs and cone beam CTs could lead to this difference [11]. Image fusion and registration, as well as precise screw planning, are important steps in the workflow of robotically assisted and navigated procedures. An error at any of these steps has the potential to decrease accuracy of the procedure. Additionally, team training, surgeon-related factors or the combination of many minor differences could also be contributing to the observed difference.

**Fig. 3:**
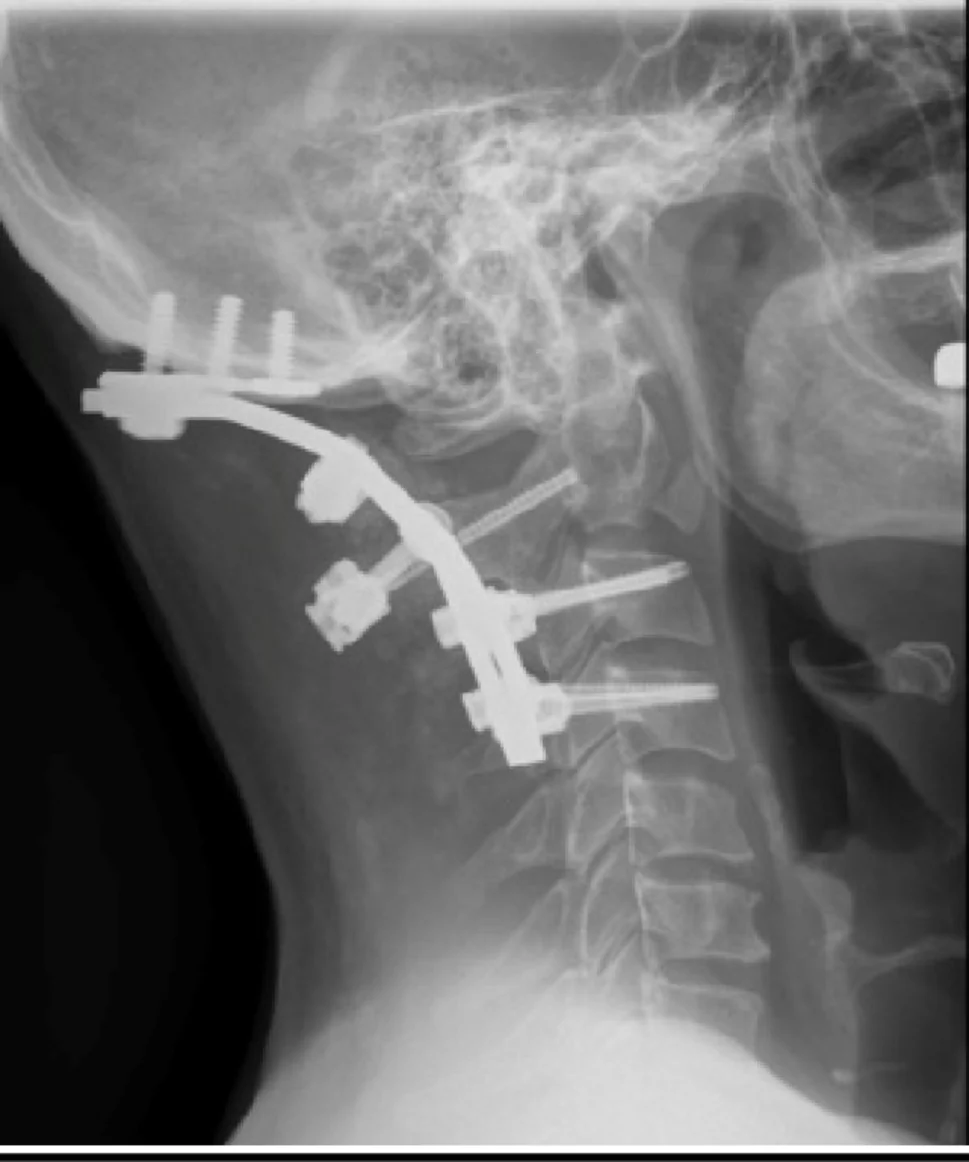
Lateral X-ray of occipitocervical fusion (C0-C2-rt.-C3-C4) for C1 ring AO type B N0 fracture (= Gehweiler III), C2 AO type B N0 and Dens type III fracture (same patient as in figures 4–9). *C*,* cervical spine*

The comparison of our data from 210 to 359 patients shows a consistently high level of accuracy. The accuracy rates at 359 patients are well within the 95% confidence intervals reported at 210 patients, so that no learning curve is visible here [11]. For our subgroup analysis of patients who underwent four-segment dorsal stabilisation without laminectomies and decompressions, for the accuracy, there is no learning curve visible either. This is likely due to the consultants’ experience with conventional and hand navigation techniques, which were developed before the implementation of the Hybrid OR. Furthermore, structured training courses, which were part of the implementation, likely contributed to the high accuracy rates from the beginning. Previous literature reports differing findings ranging from the presence of a learning curve to its absence for the accuracy rates achieved in pedicle screw placement [31, 34, 50–52]. The causes of the differences in the learning curves for the accuracy rates will be the same as those for the differing accuracy rates discussed above.

**Fig. 4:**
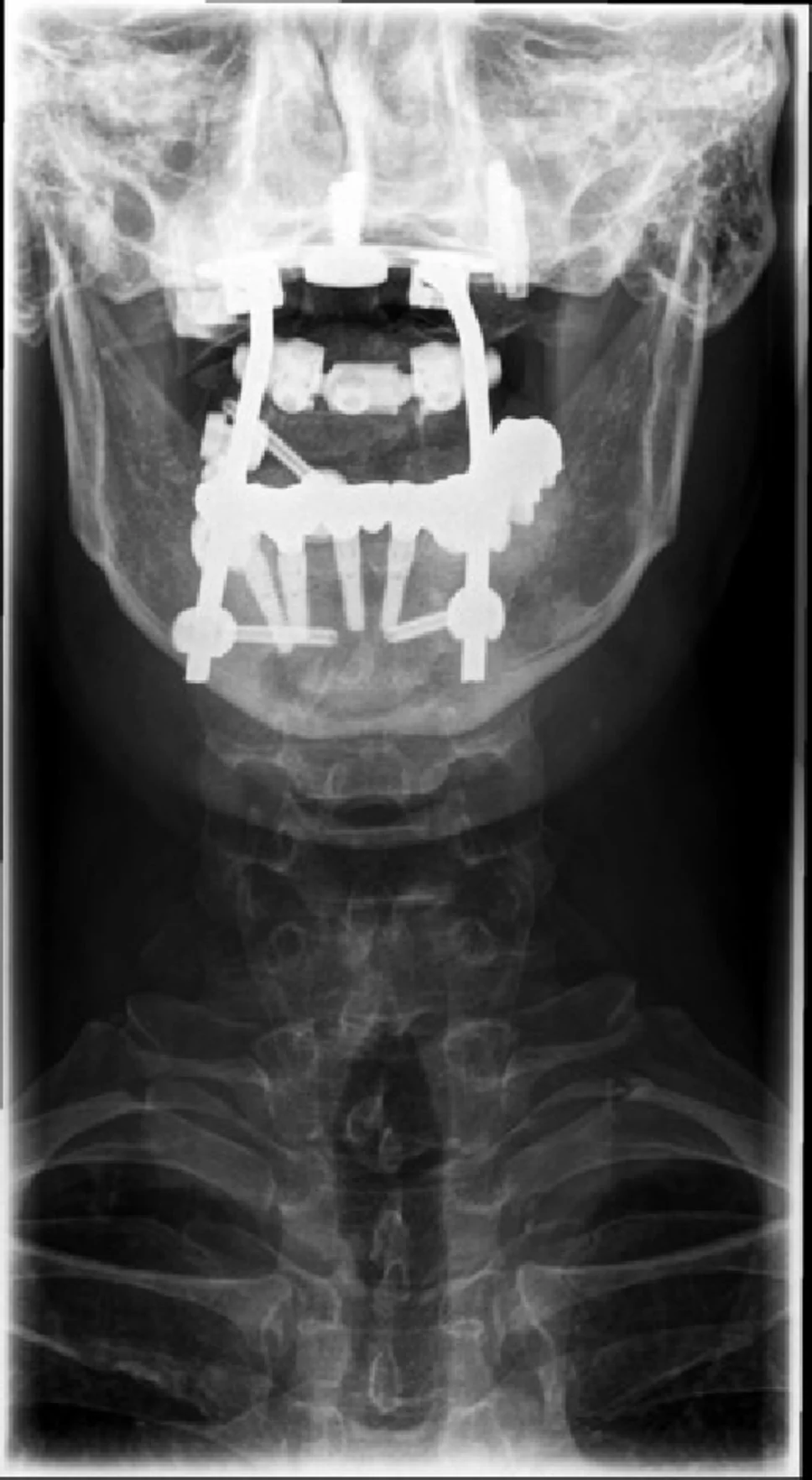
Anterior-Posterior X-ray of occipitocervical fusion (C0-C2-rt.-C3-C4) for C1 ring AO type B N0 fracture (= Gehweiler III), C2 AO type B N0 and Dens type III fracture (same patient as in figures 3 and 5–9. *C*,* cervical spine*

**Fig. 5:**
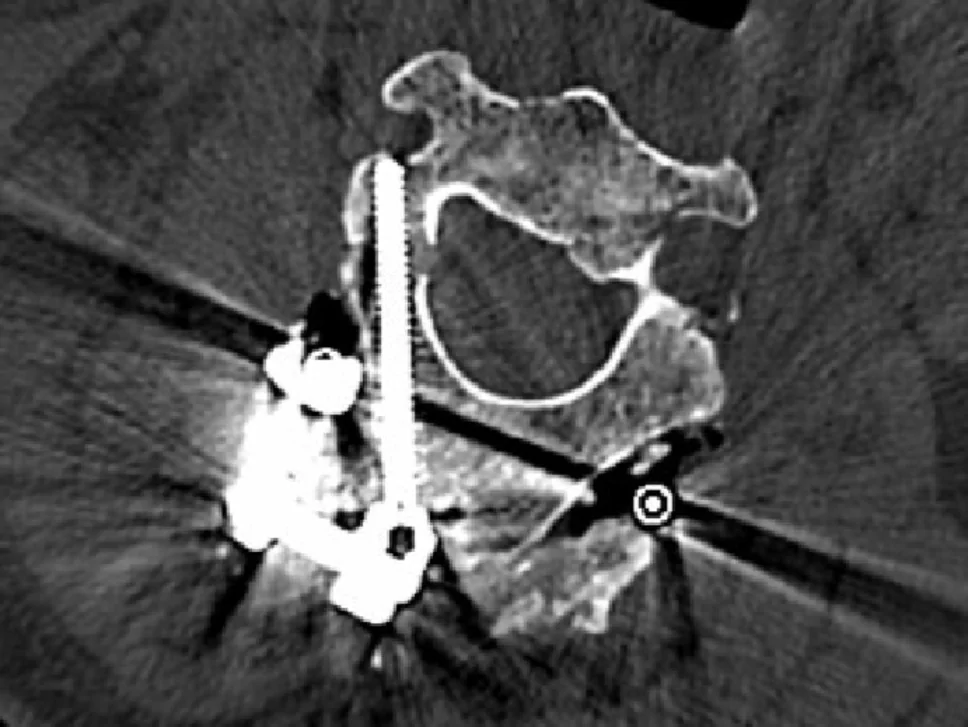
Axial CT pedicle screw C2-rt. (same patient as in figures 3–4 and 6–9). *C*,* cervical spine*

**Fig. 6:**
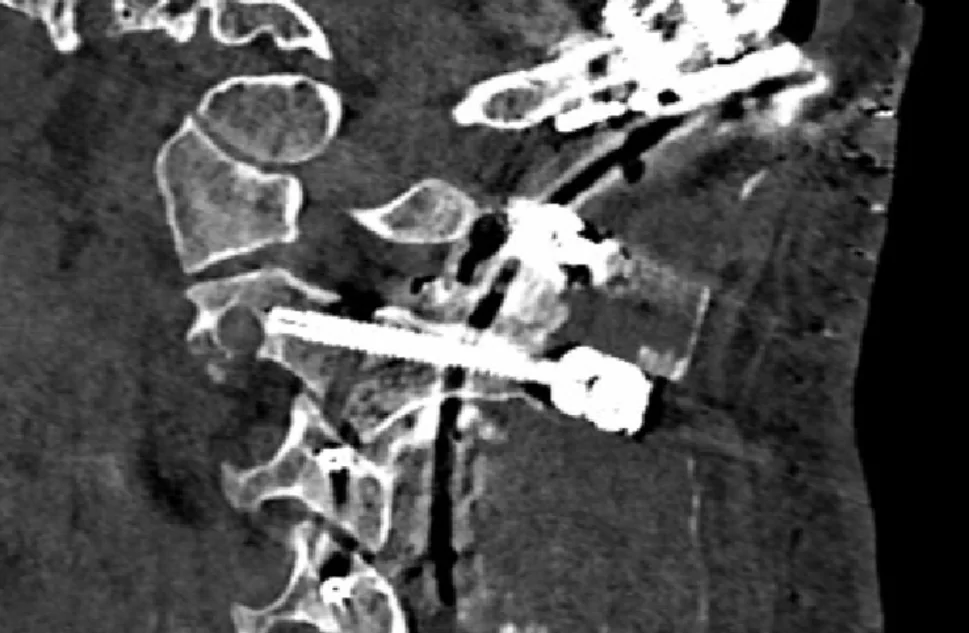
Sagittal CT pedicle screw C2-rt. (same patient as in figures 3–5 and 7–9). *C*,* cervical spine*

**Fig. 7:**
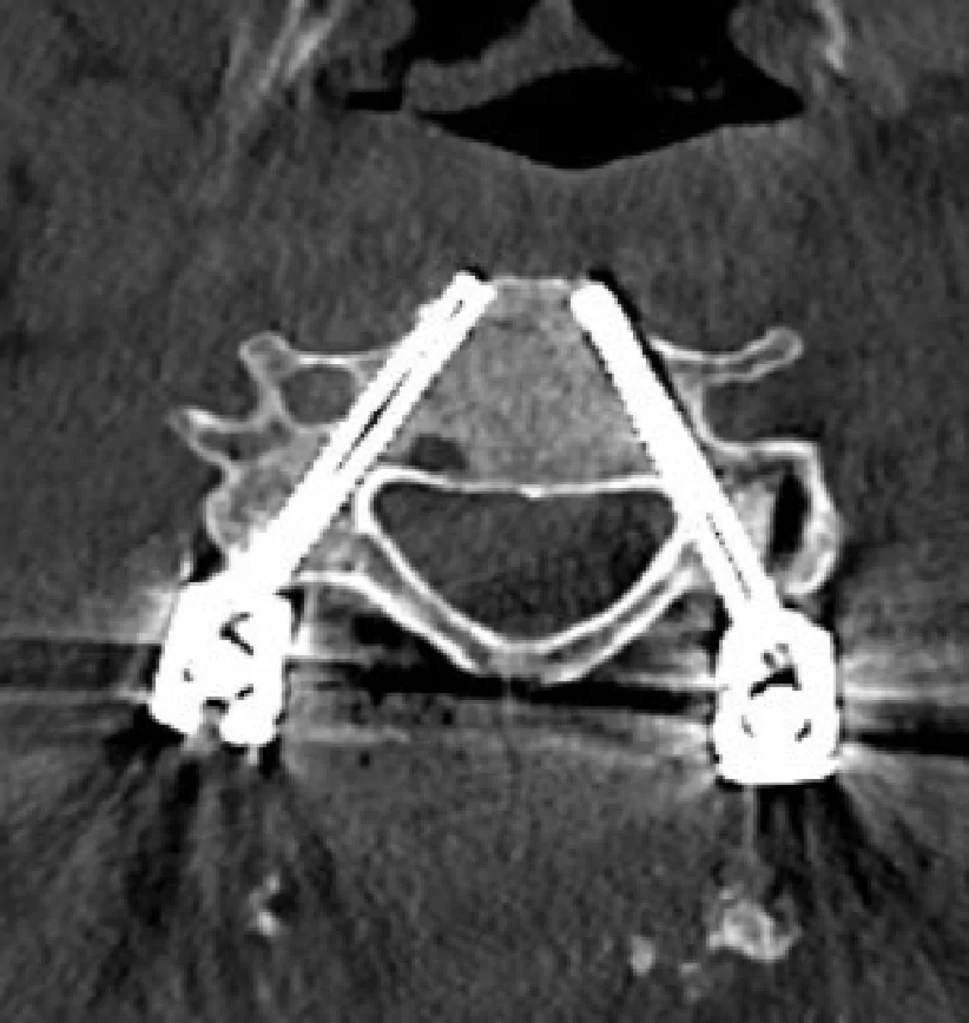
Axial CT pedicle screws C4 (same patient as in figures 3–6 and 8–9). *C*,* cervical spine*

**Fig. 8:**
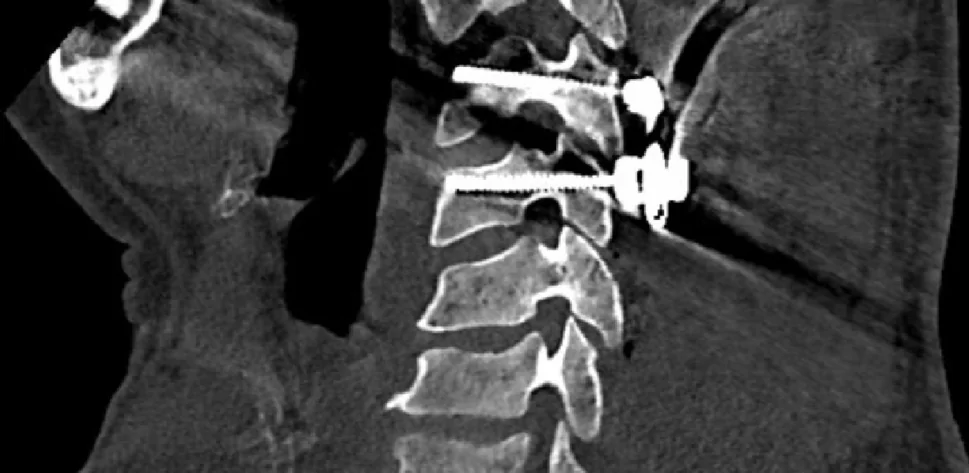
Sagittal CT pedicle screw C4-rt. (same patient as in figures 3–7 and 9). *C*,* cervical spine*

**Fig. 9:**
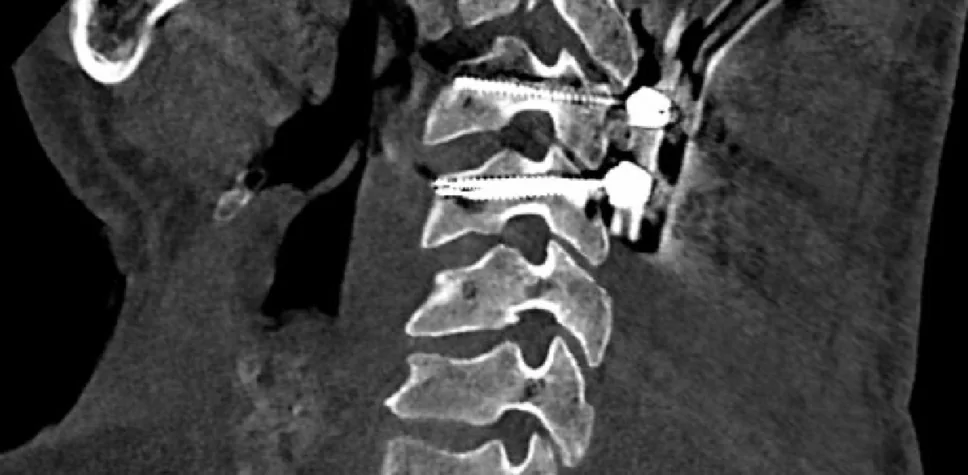
Sagittal CT pedicle screw C4-lt. (same patient as in figures 3–8). *C*,* cervical spine*

**Fig. 10:**
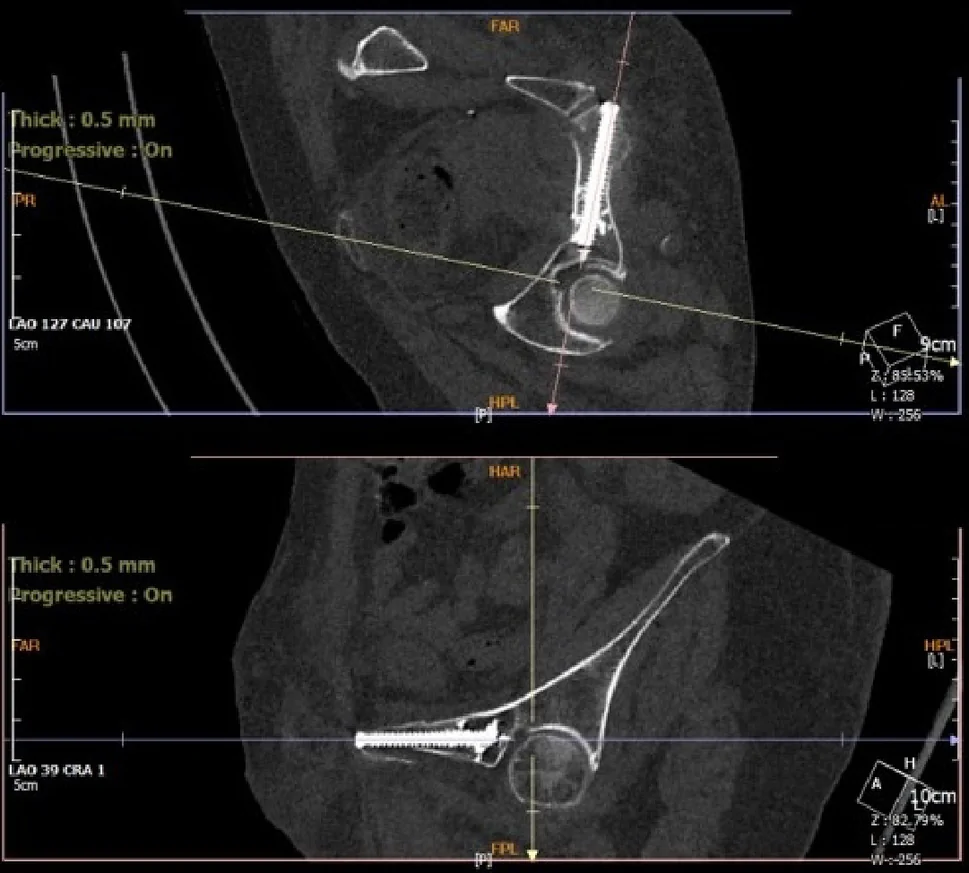
*Multiplanar CT reconstruction Pubic ramus screw lt.* (Same patient as in figures 11 and 12). Source: Haida et al. (2025), *Die Unfallchirurgie* [16]. CC BY 4.0.

**Fig. 11:**
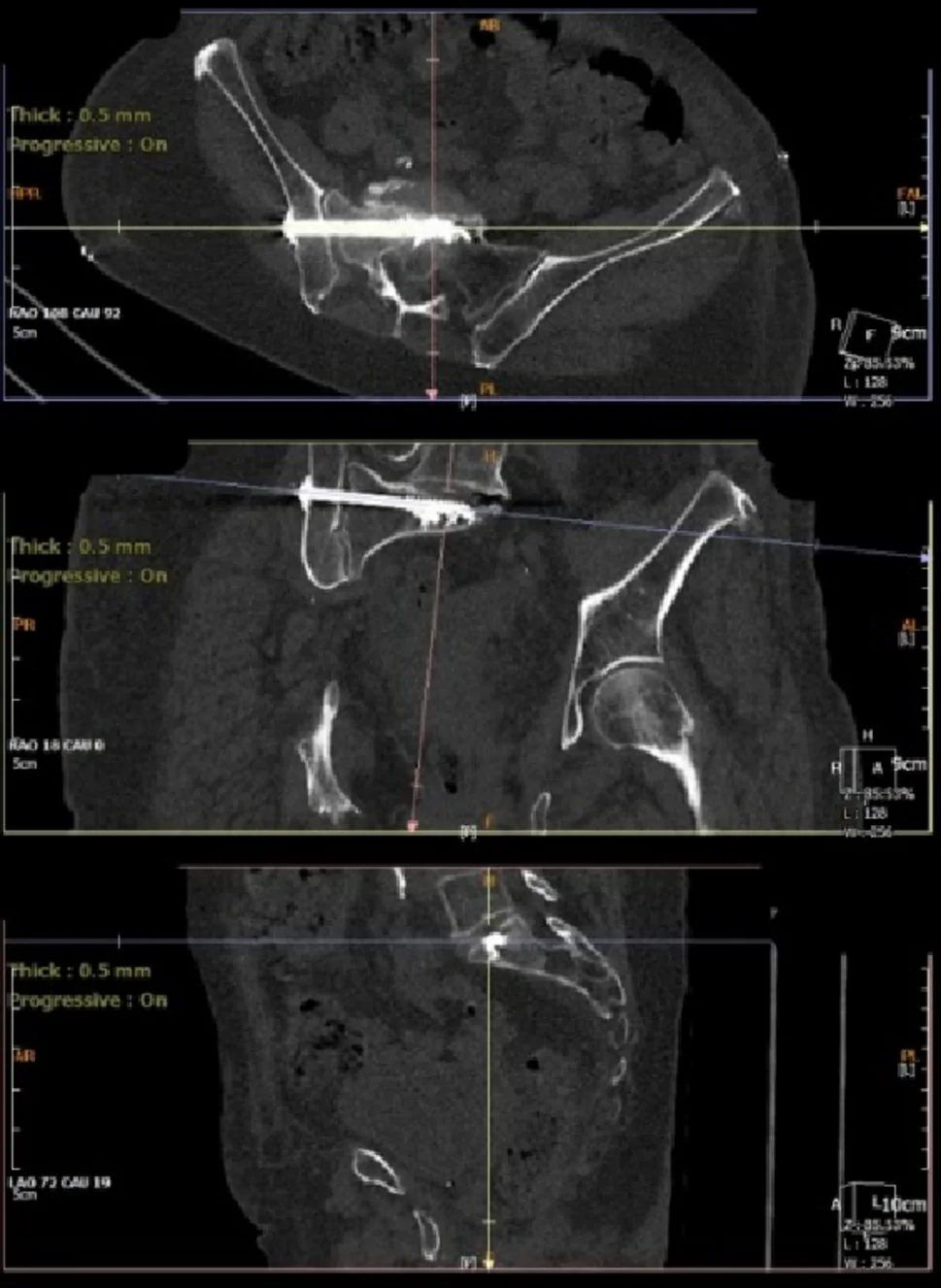
*Multiplanar CT reconstruction SI-S1-screw rt.* (Same patient as in figures 10 and 12). Source: Haida et al. (2025), *Die Unfallchirurgie* [16]. CC BY 4.0.

**Fig. 12:**
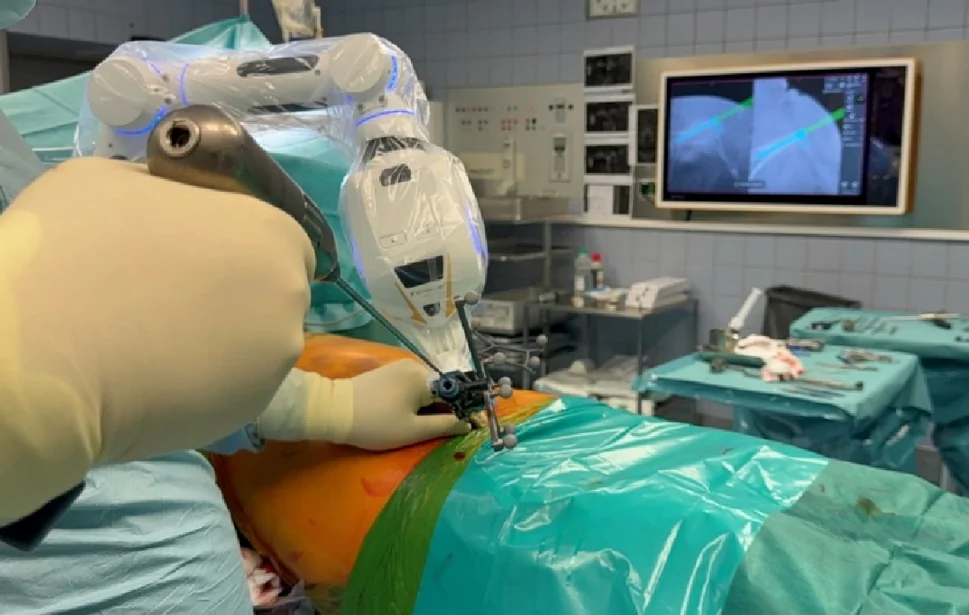
Robotically assisted screw insertion Pubic ramus screw lt. (Same patients as in figures 10 and 11). Source: Haida et al. (2025), *Die Unfallchirurgie* [16]. CC BY 4.0.

**Fig. 13:**
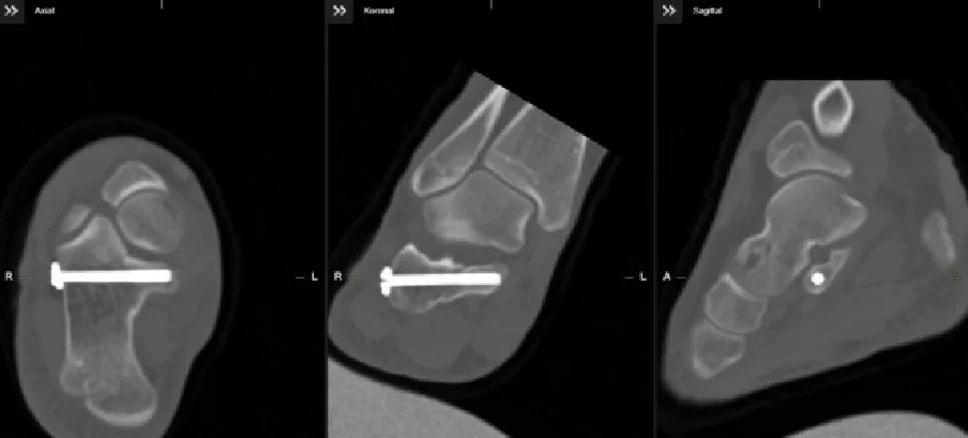
Intraoperative CBCT screw check for screw osteosynthesis of the sustentaculum tali

The findings of substantial interrater reliability are consistent with previously reported findings in literature which demonstrated similarly substantial agreement for pedicle screw assessment [21, 53–55]. When comparing these findings, differences in classification systems, use of weighted and unweighted Kappa as well as the number of reviewers and their experience should be taken into consideration, among other factors. Disagreement between reviewers can be due to the imaging quality as well as borderline cases. For the authors, the high accuracy rates achieved in this study are remarkable, as they were achieved from the implementation and have remained consistently high after more than 350 patients were treated in the Hybrid OR (Figs. 3, 4, 5, 6, 7, 8, 9, 10, 11, 12 and 13), even as the number of operating consultants increased, and new areas of interventions were established. The variability of accuracy rates reported in literature highlights to the authors that the presence of a comparable setup does not automatically result in equally high accuracy, and suggests that even small factors may have a decisive impact [23, 49].

### Limitations

There are several limitations to this study, which were already mentioned in the initial publication involving 210 patients [11]. In particular, the study design, consisting of a single-centre prospective data collection and retrospective analysis, along with the absence of a control group, limits the statistical analyses and reduces the overall value of the study. The diverse patient and intervention groups make analyses difficult. For this reason, subgroup analyses were conducted, including an analysis of the learning curve. However, these subgroup analyses are affected by distorting factors. The learning curve was analysed across multiple surgeons rather than for one individual. This means, individual experiences prior the implementation as well as the individual learning curve were not captured. Additionally, operations performed between cases belonging to the subgroups may have affected or distorted the learning curve. Overall, the statistical approach using logarithmic regression can be considered a limitation, as alternative statistical approaches are possible which may lead to different results. For the interpretation of radiation data, the inclusions of only intraoperative radiation exposure limits the assessment of radiation-related risks for patients operated in the Hybrid OR.

Screw assessment is subject to several limitations. First, it is also important to note that the grading of non-spinal screws is of a lower standard compared with the grading of spinal screws, which was carried out with an acknowledged classification system [11]. Two reviewers graded the screws without blinding to the patient’s characteristics, which could bias the evaluation of the screws. Although Cohen`s Kappa demonstrated substantial interrater reliability, this result should be interpreted with caution as it provides no information about the validity of screw grading. Furthermore, consensus-based resolution in the case of disagreements between both reviewers may have influenced the final grading results.

## Conclusion

This study demonstrates that the use of robotics and navigation in a modern Hybrid operating theatre enables consistently high accuracy rates and low complication rates over time for screw placement throughout the entire skeletal system ensuring patient safety. Compared to the previous findings at 210 patients, the accuracy rate remains similarly high despite substantial increase of operating surgeons and the introduction of new surgical procedures. Furthermore, a statistically significant learning curve was observed in the subgroup analyses with operative time decreasing over time. These findings highlight the potential of a Hybrid OR in spine and trauma surgery. Future studies should explore additional application areas and include comparison with conventional and hand navigated approaches.

## Data Availability

No datasets were generated or analysed during the current study.

## Abbreviations

AIR: Automatic Image Registration
C: Cervical spine
CBCT: Cone beam computed tomography
CDC: Centers for Disease Control and Prevention
CI95%: 95% confidence interval
CT: Computed tomography
DAP: Dose area product
DGU: Deutsche Gesellschaft für Unfallchirurgie (German Trauma Society)
DICOM: Digital imaging and communications in medicine
DWG: Deutsche Wirbelsäulengesellschaft (German Spine Society)
Et al.: et alia
GR: Gertzbein and Robbins
Gy: Gray
Hybrid OR: hybrid operating room
iCT: Intraoperative Computed Tomography
ICU: Intensive care unit
IQR: Interquartile range
ISS: Injury severity score
LN: Natural logarithm
Lt.: Left
MIS: Minimally Invasive Surgery
OR: Operating Room
RDSR: Radiation dose structure
Rt.: Right
SD: Standard Deviation
TLIF: Transforaminal lumbar interbody fusion
3D: Three dimensional
